# Substrate composition and dimensionality direct osteocyte gene expression

**DOI:** 10.1186/1753-6561-9-S7-A6

**Published:** 2015-10-27

**Authors:** Amenah Dhannoon, Robert Thomas Brady, Fergal O'Brien

**Affiliations:** 1 Royal College of Surgeons in Ireland, Dublin, Ireland

## Background

Osteoporosis has become a major public health problem, it is characterised by loss of bone mass and architecture due to disturbance in bone remodelling. Most current treatments retard bone loss but have no stimulating effect on bone formation [1]. Osteocytes act as an orchestrator for bone modelling, and secrete the glycoprotein sclerostin which negatively regulates bone formation and is a potential novel drug target [2]. Due to difficulty of accessing and studying osteocytes in vitro, an osteocyte-like MLO-Y4 cell line was developed. However, these cells only secrete sclerostin in trace amounts [3]. The objective of this study was to develop a novel biomimetic environment that would stimulate MLO-Y4 to express the osteocyte specific Sost gene.

## Methods

Four different compositions were seeded with MLO-Y4 cells and accommodated in two different cultures (3D scaffolds versus 2D films). After 5 days of culture, Sost gene expression was analysed using Real-time PCR in all groups and the data was normalised to a housekeeping gene (18s).

## Results

There was a robust statistically significant increase in MLO-Y4 gene expression for Sost when cultured on a Collagen-Hydroxyapatite (HA) substrate compared to a Collagen-only substrate. Furthermore, the 3D dimensionality enhanced gene expression across all different compositions.

## Conclusions

This study has demonstrated that scaffold composition and dimensionality has a significant influence upon regulation of MLO-Y4 gene expression. This also indicates that Sost gene is regulated by both composition and dimensionality. The ability to stimulate MLO-Y4 cell line to express Sost sufficiently will offer a precious tool for researchers to further study sclerostin secretion, identify novel regulators of Sost gene expression and investigate them to develop new therapeutic agents that may offer advantage over the currently available treatments for osteoporosis.
